# Export of Dissolved Organic Carbon from a Ponded Freshwater Marsh Receiving Diverted Mississippi River Water

**DOI:** 10.1100/tsw.2008.156

**Published:** 2008-12-14

**Authors:** R. D. DeLaune, C. B. Johnson, R. P. Gambrell, A. Jugsujinda

**Affiliations:** Department of Oceanography and Coastal Science, School of the Coast and Environment, Louisiana State University, Baton Rouge, LA 70803, USA

**Keywords:** river diversion, wetland soil, dissolved organic carbon, export, freshwater marsh, estuary

## Abstract

A series of diversion projects has been implemented to reintroduce Mississippi River water into Louisiana's coastal wetlands in order to reduce wetland loss. The export of dissolved organic carbon (DOC) was measured in a 3,700-ha ponded freshwater marsh that receives diverted Mississippi River water. Results show that highly organic marsh soil and plant material are a source of DOC. DOC, on average, was 3 mg/l greater in outlet water as compared to the concentration in river water entering the wetland. DOC in water leaving the marsh was higher in summer months, with a concentration up to 18 mg/l. Based on a discharge of 1,000 ft/sec (28.3 m/sec), it was estimated that the equivalent of 7,335 kg/day of DOC would be exported from the marsh into Lake Cataouatche, located in the northern portion of the Louisiana Barataria Basin estuary. Results suggest that river diversion would likely increase the export of DOC from the marsh as compared to normal transport associated with rainfall and tidal exchange.

### INTRODUCTION

Dissolved organic carbon (DOC) found within aquatic environments represents a broad classification of organic molecules of varied origin and composition. The "dissolved" fraction of organic carbon used by researchers represents the dissolved/colloidal fraction that passes through a 0.45-µm filter; however, 0.20 µm is also used[1]. DOC that enters water is from leaching and decomposition of soil, and sediment organic matter and plant detritus existing primarily as fulvic acids and, to a lesser extent, carbohydrate and amino acids[2].

DOC in estuaries and freshwater systems represents a significant cycled reservoir of organic matter[3]. When water exits a high organic wetland or marsh soil, a certain amount of these organic components enters adjacent stream and water bodies[4,5,6]. DOC is important in the transport of metals in aquatic systems. Metals form very strong complexes with DOC[2], enhancing metal solubility while also reducing metal bioavailability.

DOC can also result in increased growth of microorganisms in aquatic environments. The biodegradable DOC fraction (BDOC) consists of organic molecules that heterotrophic bacteria can utilize as a source of energy and carbon. Concentration of DOC in undisturbed watersheds generally ranges from approximately 1 to 20 mg/l. There is a high degree of variability in DOC concentrations across ecosystems. Wetlands are near the top of the range. The leaching of DOC from wetlands to adjacent water bodies has been well documented[4,7]. DOC in a water body is associated with the wetland area surrounding the water body[8].

Most studies have attributed export of DOC from wetlands to adjacent water bodies to be associated with runoff from rainfall leaching or tidal transport. In this study, we examined the export of DOC from a 3,700-ha ponded freshwater marsh through which Mississippi River water is diverted into the Louisiana Barataria Basin estuary.

### MATERIALS AND METHODS

#### Site Description

The Davis Pond freshwater diversion structure (one of several diversion projects) reintroduces Mississippi River water into Barataria Basin for slowing wetlands loss in the Louisiana Barataria Basin estuary. The diversion is located on the west bank of the river in St. Charles Parish, 2 mi below Luling, LA. The structure has the capacity to divert up to 10,000 $ft^3$/sec (283.2 $m^3$/sec) of the Mississippi River. The diverted waters enter through a 3,700-ha ponded freshwater marsh before entering Lake Cataouatche and, ultimately, into the Barataria Basin estuary[9]. The freshwater marsh site is dominated by a variety of freshwater vegetation including *Panicum hemitomon* Schult., *Sagittaria lancifolia* L., *Thypha spp*. L., and *Polygonum spp*. L. Soil samples (top 15 cm) at the study sites were collected using 15-cm diameter, thin-wall aluminum cylinders at the beginning of the experiment, and major soil characteristics were analyzed and are presented in Table 1 (for 3-cm increments).

Lake Cataouatche, a shallow freshwater lake located in the northern section of the Barataria Basin estuary, exhibits a mean depth of approximately 2 m and a tidal range of 0.2 m. Rainfall and nutrient runoff from adjacent upland and wetland areas are its principal inputs[10]. Lake Cataouatche is the chief receiving body of water exiting the ponded freshwater marsh/wetland.

Louisiana’s Barataria Basin is located directly south and west of New Orleans. Its boundaries are the Mississippi River to the north and east, Bayou Lafourche to west, and the Gulf of Mexico by a chain of barrier islands to the south (Fig. 1). Barataria Basin includes approximately 633,356 ha, consisting of 61,563 ha of swamp, 70,141 ha of fresh marsh, 24,076 ha of intermediate marsh, 41,571 ha of brackish marsh, and 54,068 ha of saline marsh[10,11].

#### Sampling Location

Water samples were collected for measuring change in concentrations of DOC between the inlet where diverted water first enters and at an outlet site from which water exits the ponded freshwater marsh. Additional water samples were taken from Lake Cataouatche, which receives water exiting the ponded wetland. The inflow channel, where samples were taken, was from a bridge on Hwy 90 approximately 2 mi south of Luling. This channel directs water from the Mississippi River into the 3,700-ha receiving marsh. The second site was located at the outflow from the 3,700-ha ponded wetland. Lake Cataouatche, located in the upper part of Barataria Basin, served as the third sampling site (Fig. 1).

**TABLE 1**
**Profile of Average Soil Organic Matter (OM), Mineral Content, and Bulk Density (3-cm Depth Increments) of Sediment at Four Sites within the Ponded Freshwater Marsh**

| Site | Depth (cm) | OM (%) | Mineral (%) | Bulk Density (g/cc) |
|---|---|---|---|---|
| DP-1 | 0–3 | 60.5 | 39.5 | 0.11 |
| | 3–6 | 76.4 | 23.6 | 0.08 |
| | 6–9 | 80.3 | 19.7 | 0.07 |
| | 9–12 | 80.5 | 19.6 | 0.07 |
| | 12–15 | 84.3 | 15.7 | 0.07 |
| AVE | | 76.4 | 23.6 | 0.08 |
| STDEV | | 9.3 | 9.3 | 0.02 |
| DP-2 | 0–3 | 75.1 | 24.9 | 0.04 |
| | 3–6 | 79.9 | 20.1 | 0.07 |
| | 6–9 | 76.7 | 23.3 | 0.08 |
| | 9–12 | 81.3 | 18.7 | 0.07 |
| | 12–15 | 80.9 | 19.1 | 0.08 |
| AVE | | 78.8 | 21.2 | 0.07 |
| STDEV | | 2.7 | 2.7 | 0.02 |
| DP-3 | 0–3 | 84.7 | 15.3 | 0.08 |
| | 3–6 | 82.0 | 18.0 | 0.10 |
| | 6–9 | 88.9 | 11.1 | 0.06 |
| | 9–12 | 81.5 | 18.5 | 0.10 |
| | 12–15 | 67.8 | 32.2 | 0.11 |
| AVE | | 81.0 | 19.0 | 0.09 |
| STDEV | | 7.9 | 7.9 | 0.02 |
| DP-4 | 0–3 | 88.9 | 11.1 | 0.08 |
| | 3–6 | 84.9 | 15.1 | 0.08 |
| | 6–9 | 71.4 | 28.6 | 0.08 |
| | 9–12 | 83.8 | 16.2 | 0.08 |
| | 12–15 | 79.0 | 21.0 | 0.08 |
| AVE | | 81.6 | 18.4 | 0.08 |
| STDEV | | 6.7 | 6.7 | 0.00 |

### Sample Collection

Replicate water samples were taken between September 17, 2002 and February 4, 2004 at the established sampling sites during various discharge events for comparing DOC concentration at the inlet and outlet of the ponded marsh. Flow and residence time fluctuates substantially with pulsing event. Based on a 1-m water depth during pulsing events, the estimated residence time for low discharge (10 $m^3$/sec), medium discharge (28 $m^3$/sec), and high discharge (100 $m^3$/sec) would be 42.0, 15.3, and 4.3 days, respectively. Surface water samples collected from the sample sites were taken using acid-washed high-density polyethylene bottles. All samples were stored on ice immediately on collection. Within 24 h of collection, the 350-ml samples were filtered using 0.45-µm nylon fiber filters; 40 ml was analyzed for total DOC.

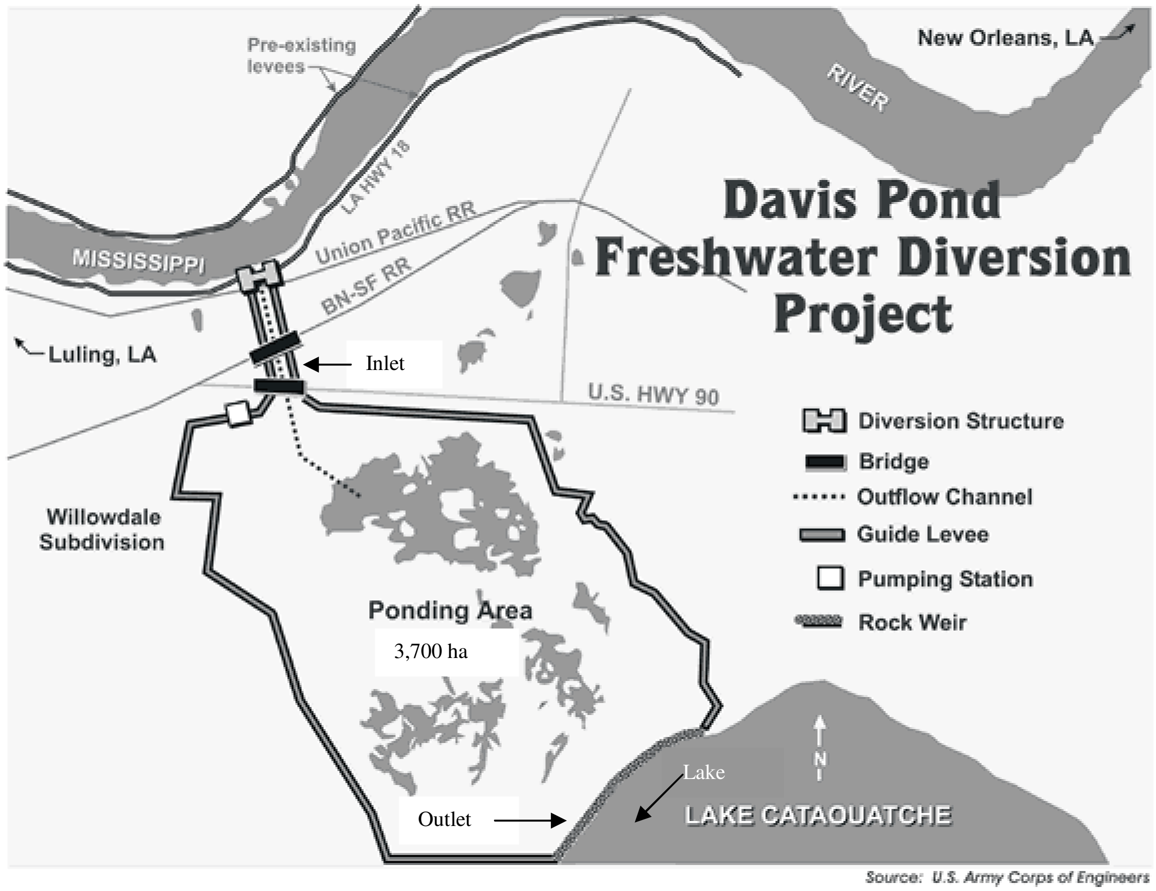

**FIGURE 1.** Sampling sites (inlet: 29°55'1.5"N, 90°19'3.1" W; outlet: 29°51'40.5"N, 90°14'2.5" W; lake: 29°51'10.5" N, 90°13'13.8" W). Adapted from Addison[10].

### Total DOC Analysis

Total DOC analysis was conducted on 40-ml samples using a carbon analyzer (Shimadzu TOC-V CSH/CSN) that is capable of measuring total inorganic, total organic, purgeable and nonpurgeable carbon, and total nitrogen. The organic carbon is oxidized to carbon dioxide, which is detected by a nondispersive infrared detector.

### Soil Organic Matter Content and Standing Plant Biomass

Bulk density and mineral content were determined in the surface 15-cm soil layer at four representative sites within the ponded freshwater marsh. Organic matter and mineral matter content was determined by loss on ignition[12]. Bulk density was determined from dry weight per unit volume of measured section (3-cm increments) of the15-cm diameter soil core collected by the aluminum cylinder. Aboveground plant material was also determined at the sample location by clipping replicated meter square plots. The vegetation was air dried and weighed.

## RESULTS

### Soil Properties/Plant Biomass

Soil organic matter content measured at four representative sites in the freshwater marsh ranged from 60 to 80% (average content in surface 0–3 cm) (Table 1). Soil bulk density ranged between 0.04 and 0.09 g/cc). Aboveground biomass at the four sites is shown in Table 2. Total (standing) aboveground biomass (live and dead) was between 75 and 550 g/m$^2$ in summer months. There was less standing biomass in the winter months, with the majority of the standing biomass being dead plant material. Both soil organic matter and aboveground plant material can serve as a source of DOC.

**TABLE 2**
**Aboveground Biomass (g/m$^2$) Determined at Four Sample Locations in 2003**

| Site | Live/Dead | Jan. 03 | Apr. 03 | Jun. 03 | Aug. 03 | Oct. 03 |
|---|---|---|---|---|---|---|
| DP1 | Live | 0 | 74 | 475 | 314 | 418 |
| | Dead | 203 | 0 | 75 | 0 | 0 |
| | Total Biomass | 203 | 74 | 550 | 314 | 418 |
| DP2 | Live | 13 | 324 | 365 | 314 | 178 |
| | Dead | 127 | 0 | 0 | 83 | 188 |
| | Total Biomass | 140 | 324 | 365 | 397 | 366 |
| DP3 | Live | 64 | 431 | 189 | 188 | 120 |
| | Dead | 281 | 0 | 0 | 202 | 354 |
| | Total Biomass | 345 | 431 | 189 | 390 | 474 |
| DP4 | Live | 0 | 87 | 76 | 370 | 354 |
| | Dead | 50 | 0 | 0 | 0 | 0 |
| | Total Biomass | 50 | 87 | 76 | 370 | 354 |

### Total DOC

Total DOC in the water samples collected at various discharge events is shown in Table 3. Samples were collected on September 17 and December 31, 2002, and on January 20, March 20, July 3, and October 8, 2003 during low discharge (less than 10 m$^3$/sec); on March 27, April 3, April 11, and April 24, 2003 during medium discharge conditions (20–40 m$^3$/sec); and on December 5 and December 12, 2003, and January 23 and February 4, 2004, during periods of high discharge (pulse reaching greater than 100 m$^3$/sec). See Fig. 2A and B for medium and high discharge rate.

Total carbon levels increased as water moved through the ponded wetland. Concentrations were higher at the outlet and in the lake when compared to those at the inlet. During the low pulse period (less than 10 m$^3$/sec), concentrations ranged from 5.5 to 10.6 mg/l at the inlet, 9.1 to 18.2 mg/l at the outlet, and 10.0 to 19.0 mg/l in the lake.

During the high pulse or discharge period, concentrations ranged from 7.4 to 8.5 mg/l at the inlet, 6.5 to 10.0 mg/l at the outlet, and 7.9 to 8.8 mg/l in Lake Cataouatche. This supports the theory that wetlands are a source for organic carbon. There was an overall trend in concentrations of increased DOC at the outflow average as presented in Table 3. Thirteen of the 14 sampling dates showed an increase in DOC at the outflow as compared to the inlet.

DOC in water exiting the ponded wetland shows an increase in DOC as compared to the inlet water, which demonstrated that the wetland system acted as a source of carbon. Summer months had the highest

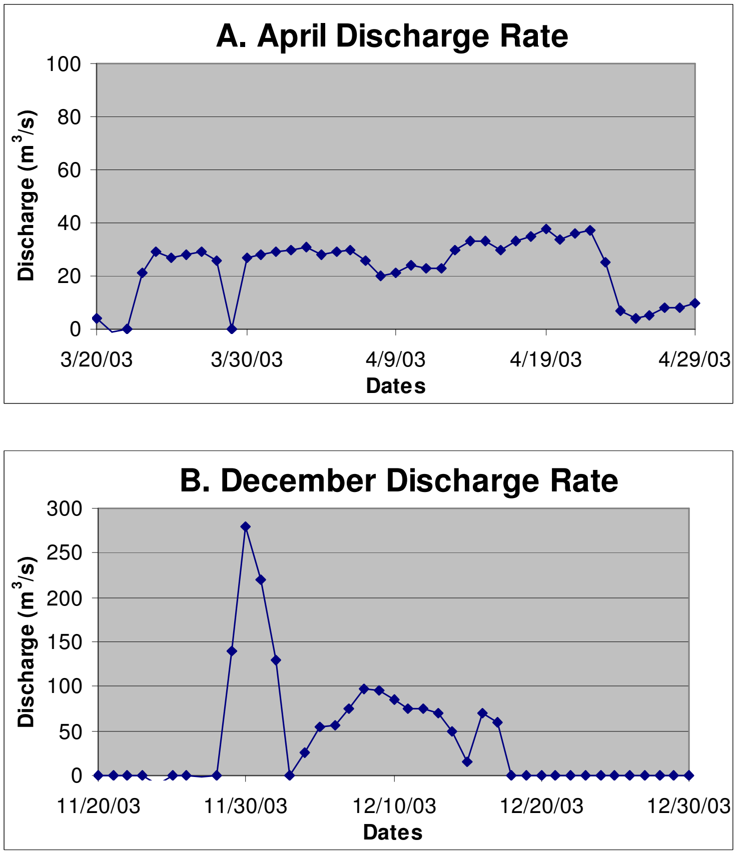

**FIGURE 2.** Discharge represents (A) medium pulse event (April) and (B) high pulse event (December).

DOC concentrations, with a concentration of approximately18.0 mg/l being observed on July 3 at the outlet. (However, it should be pointed out that there was only one summer sampling. Also, due to river stages in summer months, it is difficult to divert water into the marsh.) In contrast, spring months exhibited the lowest concentrations. The lowest outlet concentration was observed on April 3 with a concentration of approximately 5.0 mg/l. Total DOC concentrations showed smaller changes in concentration between the inlet and outlet at higher discharge rates which was attributed to dilution. On average, total DOC was approximately 3 mg/l greater in outlet water as compared to inlet water. Based on a typical discharge rate of 1,000 $ft^3$/sec (28.3 $m^3$/sec), this would be equivalent to approximately 7,335 kg/day of DOC being exported from the 3,700-ha ponded marsh during discharge events. This is small in relation to the overall carbon budget of the freshwater marsh. Future studies that more fully define flow regime and residence time would more accurately characterized DOC export. However, DOC is readily available for use by microorganisms. DOC is an important parameter due to the fact that it has the ability to influence many other functions, including the nitrogen cycle.

Similar DOC values have been reported for a riverine wetland system[14]. Surface concentration in an inflow stream ranged from 0.74 to 11.6 mg C/l and through outflow ranged from 2.1 to 8.0 mg C/l. Lu et al.[15] reported that significant amounts of dissolved organic matter (DOM) are produced in freshwater marsh sites in the southern Everglades. However, due to lack of adequate hydrological data in these reported freshwater marsh studies, it is difficult to determine amount of exported DOC from these systems. Neubauer et al.[13], in a mass balance carbon gas flux study of a tidal freshwater marsh, estimated that between 270 and 470 g/$cm^2$ of macrophyte tissue is available for deposition on the marsh surface or export from the marsh as particulate or dissolved carbon.

**TABLE 3**
**Total DOC Concentrations (mg/l) and Discharge Rates for Sampling Sites and Dates**

| Sampling Dates | | Inlet | Outlet | Lake |
|---|---|---|---|---|
| ± | 17-Sep-02 | 8.8 | 11.5 | 10.0 |
| ± | 31-Dec-02 | 5.5 | 9.1 | Not available |
| ± | 20-Jan-03 | 5.9 | 9.2 | Not available |
| ± | 20-Mar-03 | 8.8 | 11.5 | Not available |
| † | 27-Mar-03 | 4.3 | 10.5 | 14.3 |
| † | 3-Apr-03 | 4.3 | 5.2 | 9.8 |
| † | 11-Apr-03 | 3.8 | 5.8 | 8.1 |
| † | 24-Apr-03 | 3.8 | 5.3 | 7.9 |
| ± | 3-Jul-03 | 8.5 | 18.2 | 17.1 |
| ± | 8-Oct-03 | 10.6 | 18.1 | 19.0 |
| ‡ | 5-Dec-03 | 7.8 | 9.1 | 7.9 |
| ‡ | 12-Dec-03 | 8.5 | 10.0 | 8.8 |
| ‡ | 23-Jan-04 | 7.5 | 8.4 | 8.1 |
| ‡ | 4-Feb-04 | 7.4 | 6.5 | 8.0 |
| Average | | 6.8 | 9.9 | 10.8 |
| Standard Deviation | | 2.2 | 4.1 | 4.1 |

± Low pulse, † medium pulse, ‡ high pulse.

The flux of organic carbon and inorganic nutrients between intertidal marsh to estuarine water has been studied for determining influence on primary and secondary productivity. In estuaries, studies have suggested that there must be a significant source of DOC from marsh to estuarine water to account for measured supersaturated levels of $CO_2$[16,17,18,19]. Neubauer and Anderson[20], in a study of a tidal freshwater marsh and adjacent estuary, documented an export of DOC from the marsh into the York River estuary. When the surface of a tidal marsh is flooded, the upward diffusion of marsh pore water and the decomposed plant material and detritus material on the marsh surface are the primary source of DOC to the water column[20]. Schindler et al.[7] reported that DOC concentration doubled as water passed through a small bog lake and also increased with flooding of a wetland area around the edge of the lake.

In a study of DOC in East and Gulf Coast Estuaries, Peterson et al.[1] reported that riverine inputs generally had higher DOC concentrations than coastal or saline environments. Riverine DOC varied from approximately 4 to 12 mg/l compared to 2.4 mg/l at a saline site near the coast.

The ponded freshwater marsh in this study has previously been shown to be efficient in the removal of nitrate from the diverted Mississippi River water. Practically all the nitrate found in the river water was removed primarily through denitrification[9,21]. Water exiting the ponded freshwater marsh has been shown to be higher in ammonium-N than water entering the marsh[22]. The previous studies did not distinguish between denitrification and dissimilatory nitrate reduction to ammonium, which could account for some of the measured ammonium-N export.

### CONCLUSIONS

This study clearly shows that DOC concentration in water leaving the freshwater wetland was elevated in relation to water entering the ponded wetland from the Mississippi River. Water exiting the freshwater wetlands supplies a significant amount of DOC to Lake Cataouatche that would likely be readily available to bacteria. Since microbial activity in estuarine water depends on DOC to a large degree, estuarine productivity depends heavily on the source of DOC from adjacent wetland ecosystems. Even though this study reflects DOC export from a ponded region receiving pulses of Mississippi River water, the results of this study would also suggest export of DOC associated with rainfall runoff from the 70,141 ha of freshwater marshes located in Barataria Basin. However, results also suggest that river diversion or the reintroduction of Mississippi River into Louisiana's coastal wetland to slow or recover wetland loss would also increase the export of DOC to the lower estuaries as compared to normal transport associated with rainfall and tidal exchange.

### ACKNOWLEDGMENT

The research was funded in part by the Louisiana Department of Natural Resources.
